# Prevalence and Correlates of *Cryptosporidium* Infections in Kenyan Children With Diarrhea and Their Primary Caregivers

**DOI:** 10.1093/ofid/ofaa533

**Published:** 2020-10-31

**Authors:** Emily L Deichsel, Heidi K Hillesland, Carol A Gilchrist, Jaqueline M Naulikha, Christine J McGrath, Wesley C Van Voorhis, Doreen Rwigi, Benson O Singa, Judd L Walson, Patricia B Pavlinac

**Affiliations:** 1 University of Maryland, Baltimore, Maryland, USA; 2 Hawai’i Pacific Health, Lihue HI; 3 University of Virginia, Charlottesville, Virginia, USA; 4 Maasai Mara University, Narok, Kenya; 5 University of Washington, Seattle, Washington, USA; 6 Kenya Medical Research Institute, Nairobi, Kenya; 7 Child Acute Illness and Nutrition (CHAIN) Network, Nairobi, Kenya

**Keywords:** caregiver, *Cryptosporidium*, diarrhea, infant, transmission

## Abstract

**Background:**

*Cryptosporidium* is a leading cause of diarrhea in Sub-Saharan Africa and is associated with substantial morbidity and mortality in young children.

**Methods:**

*** ***We analyzed data from children aged 6–71 months presenting to 2 public hospitals in Western Kenya with acute diarrhea and their primary caregivers, including detection of *Cryptosporidium* by quantitative polymerase chain reaction (PCR) and immunoassay analysis in stool samples from both children and their caregivers. Associations between potential transmission sources and child/caregiver *Cryptosporidium* infection were evaluated using prevalence ratios (PRs). Secondary analyses evaluated host and clinical risk factors of child/caregiver *Cryptosporidium* infection.

**Results:**

Among 243 child–caregiver pairs enrolled, 77 children (32%) and 57 caregivers (23%) had *Cryptosporidium* identified by either immunoassay or PCR. Twenty-six of the 243 child–caregiver pairs (11%) had concordant detection of *Cryptosporidium*. *Cryptosporidium* infection in children was associated with detection of *Cryptosporidium* in caregivers (adjusted PR [aPR], 1.8; 95% CI, 1.2 to 2.6; *P = *.002) and unprotected water source (aPR, 2.0; 95% CI, 1.3 to 3.2; *P = *.003). Risk factors for *Cryptosporidium* detection in caregivers included child *Cryptosporidium* infection (aPR, 2.0; 95% CI, 1.3 to 3.0; *P = *.002) as well as cow (aPR, 3.1; 95% CI, 1.4 to 7.0; *P = *.02) and other livestock ownership (aPR, 2.6; 95% CI, 1.1 to 6.3; *P = *.03) vs no livestock ownership. Recent diarrhea in caregivers and children was independently associated with child and caregiver *Cryptosporidium* infections, respectively.

**Conclusions:**

Our results are consistent with the hypothesis that *Cryptosporidium* transmission can occur directly between child–caregiver dyads as well as through other pathways involving water and livestock. Additional research into caregivers as a source of childhood *Cryptosporidium* infection is warranted.

*Cryptosporidium* is a leading cause of diarrhea among children in many resource-limited settings [1, 2]. *Cryptosporidium* is responsible for >60_ _000 child deaths per year [3] and is also associated with linear growth faltering [4–6]. No vaccine is available to prevent *Cryptosporidium,* and current treatment options are limited, particularly for children with malnutrition or HIV, conditions common in Sub-Saharan Africa [7, 8]. An improved understanding of C*ryptosporidium* epidemiology and transmission dynamics may illuminate opportunities for interventions to reduce C*ryptosporidium*-associated morbidity and mortality in young children.

*Cryptosporidium* is transmitted person-to-person (anthroponotic transmission) [9, 10], through contact with infected animals (zoonotic) [10], and through ingestion of contaminated water [11]. The predominance of anthroponotically transmitted subtypes of *Cryptosporidium* observed in Sub-Saharan Africa suggests that person-to-person transmission may be a predominant transmission pathway in this region [10, 12].

Caregivers may be a source of *Cryptosporidium* infection in young children or may have secondary infections from children or other close contacts. In Western Kenya, caregiver HIV infection, a known risk factor of *Cryptosporidium* susceptibility and prolonged oocyst shedding [13, 14], was associated with child *Cryptosporidium* infection [15], even in the absence of child HIV infection. In addition, other factors such as childhood malnutrition, breastfeeding history, and environmental factors may dramatically affect risk of child *Cryptosporidium* infection [5, 6, 16].

Among children presenting with acute diarrhea at 2 hospitals in Western Kenya, we sought to determine the prevalence of *Cryptosporidium* in accompanying primary caregivers and to identify risk factors for infections in both the children and their caregivers.

## METHODS

### Study Design

Between March and December 2015, children aged 6 to 71 months presenting with acute diarrhea (2 or more loose stools per 24-hour period and lasting <7 consecutive days) to 2 public hospitals in Western Kenya (Kisii Teaching and Referral Hospital and Homa Bay County Referral Hospital) were enrolled in this cross-sectional study. Children were excluded if they were not accompanied by a biological parent or legal guardian, if they were unable to provide a stool sample, or if the primary caregiver elected not to receive HIV counseling and testing (if indicated). Sociodemographic characteristics, breastfeeding history, and clinical history of the child and caregiver were collected by a standardized questionnaire, and a brief physical exam of the child was performed by study clinicians. Study staff measured height (or length if <2 years) and weight and assessed danger and dehydration signs according to the World Health Organization (WHO) Integrated Management of Childhood Illness (IMCI) algorithm. Children were managed according to Kenyan Ministry of Health guidelines for diarrhea [17]. Height/length for age and weight for height/length z-scores (HAZ and WHZ) were calculated for children using WHO reference populations, and stunting and wasting were defined as HAZ and WHZ <–2, respectively [18, 19]. A body mass index (BMI) <18.5 kg/m^2^ in adult caregivers was defined as underweight. Moderate to severe diarrhea (MSD) was defined as diarrhea with signs of dehydration (sunken eyes, loss of skin turgor, intravenous hydration administered or prescribed), visible blood in stool, or hospital admission based on diarrhea or dysentery, as defined elsewhere [20].

A whole stool sample was collected from enrolled children before administration of antibiotic therapy or hospital admission (if applicable). Caregivers were asked to provide a stool sample at the hospital before returning home. Stool samples were accepted from caregivers up to 72 hours after child enrollment. After collection, stool samples were immediately placed in a cool box and maintained at 2–8°C until further processing.

### Laboratory Methods

Stool was processed within 2 hours of receipt of the sample. A small portion of the stool sample was used for immediate *Cryptosporidium* testing using a point-of-care immunoassay (Quik Chek, Alere). The Quik Chek enzyme-linked immunosorbent assay (ELISA) was used in accordance with the manufacturer’s instructions [21]. The remaining stool sample was aliquoted into 2-mL cryovial storage containers and frozen to –80°C within 36 hours of enrollment. DNA extraction of stool samples was performed using a QiaAmp stool DNA extraction protocol that included bead-beating for oocyst lysing, and extracted DNA was shipped to the University of Virginia for quantitative PCR using previously described methods [22].

A small amount of blood (<0.5 mL) was collected from caregivers if there was no documentation of HIV status in the last 2 months. If the biological mother was HIV-infected or had an unknown HIV status, blood was collected either by heel or finger prick from the child. Adults and children over 18 months of age were tested for HIV using antibody testing (Abbott Determine rapid test kit) and confirmed using First Response (Premier Medical Corporation). Both tests were performed according to the manufacturer’s instructions. HIV DNA PCR assays were performed in children <18 months of age. Children of known HIV-infected biological mothers who were HIV-uninfected themselves were classified as HIV-exposed uninfected (HEU).

### Statistical Analysis

*Cryptosporidium* infection was defined as detection of *Cryptosporidium* antibodies (by immunoassay) or DNA (by PCR at the lower limit of detection [cycle threshold {CT} values ≤40]).

Univariate prevalence ratios (PRs) estimating the association between child and caregiver *Cryptosporidium* infections were estimated using Poisson regression with robust variance estimates [23]. Multivariable models included detection of *Cryptosporidium* in children and caregivers, as defined above, as well as other potential transmission sources (water, livestock, inadequate sanitation). As the primary aim of the analysis was to determine potential sources of *Cryptosporidium* infection, host and clinical characteristics were not included in the primary analyses. However, in exploratory analyses, we examined general host and clinical risk factors for *Cryptosporidium* infection, separately in children and caregivers, using Poisson regression with robust variance estimates, respectively. Separate multivariable models were constructed for each risk factor, each adjusting for recruitment site as well as child and caregiver age (in child and caregiver models, respectively). Student *t* tests were used to assess mean CT values by immunoassay result. All analyses were performed using Stata 15.1 (College Station, TX, USA), with an alpha of .05 used to determine statistical significance.

### Minimum Detectable Effect

We calculated the minimal detectable effect for analyses examining risk factors for child *Cryptosporidium* infection. Using our sample of 243 participants, we assumed that 31% of children presenting to a health care facility with diarrhea would have *Cryptosporidium* detected by ELISA or PCR. Assuming 10%, 25%, or 50% of participants without *Cryptosporidium* detection have the risk factor, we had 80% power to detect a prevalence ratio of 2.4, 1.8, and 1.4, respectively.

### Patient Consent Statement

The study was approved by the Kenya Medical Research Institute Ethics and Research Committee and the University of Washington Institutional Review Board. Written informed consent was obtained from all primary caregivers for their and their child’s participation in the study.

## RESULTS

### Study Population

**Figure 1.**
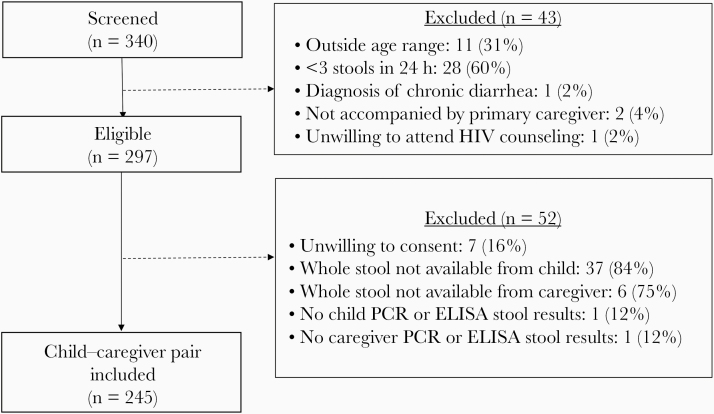
Eligibility flowchart. Abbreviations: ELISA, enzyme-linked immunosorbent assay; PCR, polymerase chain reaction.

Two hundred forty-three children with acute diarrhea and their caregivers were enrolled in the study (Figure 1). Fifty-three percent (129) of the children were enrolled from Kisii County Hospital, and 94% (227) were accompanied by their biological mother (Table 1). Forty-six percent (112) of the enrolled children were under 2 years of age. Of the enrolled children, 28% (68) had MSD and 27% (66) reported fever at the time of presentation. Two children (0.8%) were HIV-infected, and 26 (11%) were HEU. Caregivers were a median (interquartile range [IQR]) of 28 (23–32) years of age, 12% (28) were HIV-infected, and 3% (7) were underweight. Eight percent (20) of caregivers reported diarrhea in the previous 14 days.

**Table 1.** Characteristics of Enrolled Children With Acute Diarrhea and Their Primary Caregiver (n = 243 Child–Caregiver Pairs)

Characteristic	n Median	(%) or (IQR)
Sociodemographic		
Site		
Kisii	129	(53.1)
Homa Bay	114	(46.9)
Child accompanied by biological mother	227	(93.4)
Monthly household income <5000 Kenyan shillings^a^	76	(33.5)
Any livestock ownership	178	(73.3)
Cows	117	(48.2)
Chickens	168	(69.1)
Goats/sheep	64	(26.3)
Owns the land they currently live on	183	(75.3)
Toilet type		
Pit latrine	219	(90.1)
Flush toilet	20	(8.2)
Open defecation	4	(1.7)
Shared toilet^b^	163	(68.2)
Unimproved flooring^c^	84	(34.6)
Persons per room	2	(1.3–3.0)
Household crowding (≥2 people per room)	129	(53.1)
No. of children <59 mo in household	1	(1–2)
Unprotected water source^d^	18	(7.4)
Water source shared with >1 household	223	(91.8)
Reports treating drinking water^e^	176	(72.4)
Boiling	80	(32.9)
Filtration	5	(2.1)
Treat with bleach/chlorine	91	(37.5)
Straining through a cloth	3	(1.2)
Let it stand and settle	12	(4.9)
Child characteristics and clinical presentation/history		
Female	120	(49.4)
Age, mo	25	(13–37)
6–11	51	(21.0)
12–23	61	(25.1)
24–71	131	(53.9)
≥1 IMCI danger sign	62	(25.5)
Moderate to severe diarrhea^f^	68	(28.0)
Evidence of dehydration	41	(16.9)
None	202	(83.1)
Some	30	(12.4)
Severe	11	(4.5)
Duration of diarrhea, d	2	(2–3)
No. of loose stools in last 24 h	4	(4–6)
Stunting (HAZ <–2)^g^	51	(21.6)
Wasting (WHZ <–2)^h^	31	(13.3)
MUAC <12.5 cm	15	(6.2)
Malaria^i^	20	(8.4)
Febrile (axillary temperature ≥37.5°C)	66	(27.2)
Recent antimicrobial use	51	(21.0)
Child ever breastfed	237	(97.5)
Currently breastfeeding (among <24-mo-olds)^j^	79	(70.5)
Reported to be exclusively breastfed for 6+ mo^k^	181	(78.7)
HIV infected^l^	2	(0.8)
HIV-exposed uninfected^m^	26	(10.9)
Caregiver characteristics and clinical history		
Age, y	28	(23–32)
BMI, kg/m^2^	24.0	(22.0–27.4)
BMI <18.5 kg/m^2^ (or underweight)	7	(2.9)
Diarrhea within past 14 d	20	(8.2)
Vomiting within past 14 d	7	(2.9)
Hospitalized in the last y	13	(5.4)
HIV-infected^n^	28	(11.7)
On HAART	13	(46.4)
Not on HAART	15	(53.6)
Recent antimicrobial use	9	(3.7)

Abbreviations: HARRT, highly active antriretroviral therapy; BMI, body mass index; HAZ, height-for-age z-score; IMCI, Integrated Management of Childhood Illness; IQR, interquartile range; MUAC, mid-upper arm circumference; WHZ, weight-for-height z-score.

^a^Among 227 who answered the income question.

^b^Among 239 who answered the shared toilet question.

^c^Dirt floor.

^d^Unprotected well, tubewell, borehole, rainwater, surface water.

^e^Categories not mutually exclusive.

^f^One or more of the following: sunken eyes, loss of skin turgor, intravenous hydration administered or prescribed, visible blood in stool, or hospital admission based on diarrhea or dysentery [20].

^g^Among 236 children with measured length.

^h^Among 234 children with measured weight and length.

^i^Among 239 children with malaria test results.

^j^Among 112 children with under 24 months of age.

^k^Among 230 who answered breastfeeding duration question.

^l^Among 241 children with known HIV status.

^m^Among 238 HIV-uninfected children in whom maternal HIV status was known.

^n^Among 240 caregivers with known status.

Seventy-three percent (178) owned some type of livestock, with chickens being the most common (168), followed by cows (117). Ninety percent (219) also reported access to pit latrines (vs flush toilet or open defecation). Eighteen (7%) households reported having access to only unprotected water sources (unprotected well, tubewell, borehole, rainwater, surface water). Most caregivers did not report treating their drinking water with methods effective against *Cryptosporidium* oocyst contamination, boiling or filtering water (33% of caregivers reported boiling, and 2% reported filtering).

### Detection of Cryptosporidium

Seventy-seven children (32%; 95% CI, 26% to 38%) had *Cryptosporidium* identified by either immunoassay or PCR, as did 57 (23%; 95% CI, 19% to 29%) caregivers. Among the 77 children with *Cryptosporidium* infection, *Cryptosporidium* was detected in 27 (35%; 95% CI, 25% to 46%) caregivers. The median CT values (IQR) among children and caregivers with positive detections by PCR were 35.1 (31.4–36.5) and 36.2 (35.3–37.2), respectively. Among children and caregivers with both immunoassay and PCR results available, the immunoassay captured 20% (15/76) and 4% (2/54) of PCR-detected *Cryptosporidium*, and the overall percent agreement of the 2 tests was 73% (162/223) and 77% (181/236), respectively. Two positive immunoassay tests for caregivers were negative by PCR (Table 2). In children with *Cryptosporidium* identified by PCR, those with a positive immunoassay result had a higher parasitic burden (lower CT values), on average, than children with a negative immunoassay result (26.0 vs 35.1; mean difference, 9.2; 95% CI, 7.3 to 11.0; *P* < .001). No difference in mean CT values was observed between caregivers with positive and negative immunoassay results (37.4 vs 35.7; mean difference, –1.7; 95% CI, –5.4 to 2.1; *P = *.367).

**Table 2.** Concordance of *Cryptosporidium* Identified by PCR and ELISA Among Enrolled Children and Primary Caregivers With Both ELISA and PCR Results (n = 243 Child–Caregiver Pairs)

Child	PCR Positive	PCR Negative	PCR Missing
ELISA positive	15	0	1
ELISA negative	61	147	19
Adult			
ELISA positive	2	2	0
ELISA negative	53	179	7

Abbreviations: ELISA, enzyme-linked immunosorbent assay; PCR, polymerase chain reaction.

### Risk Factors for Child Cryptosporidium

Risk factors for child *Cryptosporidium* infection included detection of *Cryptosporidium* among their caregiver and using an unprotected water source (Table 3). Children with a *Cryptosporidium-*infected caregiver were nearly 2 times more likely to have *Cryptosporidium* themselves (adjusted prevalence ratio [aPR], 1.8; 95% CI, 1.2 to 2.6; *P = *.002). In addition, living in a household with an unprotected water source was significantly associated with detection of *Cryptosporidium* in a child (aPR, 2.0; 95% CI, 1.3 to 3.2; *P = *.003). Livestock ownership did not appear to be an important predictor for child *Cryptosporidium* infections.

**Table 3.** Transmission Sources of *Cryptosporidium* Among Enrolled Children and Primary Caregivers (n = 243)

	Child *Cryptosporidium* Detection^a^				Caregiver *Cryptosporidium* Detection^b^			
	Prevalence Ratio (95% CI)	*P* Value	Adjusted^c^ Prevalence Ratio (95% CI)	*P* Value	Prevalence Ratio (95% CI)	*P* Value	Adjusted^d^ Prevalence Ratio (95% CI)	*P* Value
Unprotected water source	2.1 (1.4 to 3.2)	.001	2.0 (1.3 to 3.2)	.003	1.5 (0.7 to 3.0)	.28	0.8 (0.4 to 1.9)	.67
Household filters or boils water	0.9 (0.6 to 1.3)	.58	1.0 (0.6 to 1.4)	.80	1.0 (0.6 to 1.6)	.98	1.0 (0.6 to 1.6)	.99
Shared toilet^e^	1.1 (0.7 to 1.7)	.59	1.1 (0.7 to 1.7)	.62	0.8 (0.5,1.2)	.24	0.8 (0.5 to 1.3)	.34
Livestock ownership								
None	Ref	–	Ref	–	Ref	–	Ref	--
Livestock other than cow	1.4 (0.9 to 2.3)	.17	1.2 (0.7 to 2.1)	.41	3.0 (1.3 to 7.2)	.01	2.6 (1.1 to 6.3)	.03
Cows	1.1 (0.7 to 1.7)	.75	0.8 (0.5 to 1.4)	.53	3.1 (1.4 to 7.1)	.01	3.1 (1.4 to 7.0)	.01
Child *Cryptosporidium* infection	NA	–	NA	–	1.9 (1.2 to 3.0)	.004	2.0 (1.3 to 3.1)	.003
Caregiver *Cryptosporidium* infection	1.8 (1.2 to 2.5)	.001	1.8 (1.2 to 2.6)	.002	NA	–	NA	–

Abbreviation: HAART, highly active antiretroviral therapy.

^a^Total 77 child *Cryptosporidium* detections used.

^b^Total 57 caregiver *Cryptosporidium* detections used.

^c^Multivariable model includes unprotected water source, method of water safety improvement, shared toilet, livestock ownership, and presence of caregiver *Cryptosporidium* (n = 239).

^d^Multivariable model includes unprotected water source, method of water safety improvement, shared toilet, livestock ownership, and presence of child *Cryptosporidium* (n = 239).

^e^n = 239.

Age, HIV infection and HIV exposure status, malnutrition, breastfeeding history, and severity of diarrhea were not associated with child *Cryptosporidium* infection in adjusted analyses (Table 4). However, younger caregiver age and caregiver-reported diarrhea were important for child *Cryptosporidium* infection. Children of caregivers <20 years of age were twice as likely to have *Cryptosporidium* detected than children with caregivers aged 20–29 years (aPR, 2.0; 95% CI, 1.3 to 3.1; *P* = .002). In addition, children of caregivers reporting diarrhea within the last 2 weeks were twice as likely to have *Cryptosporidium* themselves (aPR, 2.2; 95% CI, 1.3 to 3.4; *P* = .001). Caregiver HIV and BMI were not associated with child *Cryptosporidium* infection.

**Table 4.** Host and Clinical Correlates of *Cryptosporidium* Infections Among Enrolled Children and Primary Caregivers (n = 243)

	Child *Cryptosporidium* Detection^b^				Caregiver *Cryptosporidium* Detection^c^			
	Prevalence Ratio (95% CI)	*P* Value	Adjusted^d^ Prevalence Ratio (95% CI)	*P* Value	Prevalence Ratio (95% CI)	*P* Value	Adjusted^e^ Prevalence Ratio (95% CI)	*P* Value
Child characteristics								
Age								
6–12 mo	0.9 (0.6 to 1.5)	.66	0.9 (0.6 to 1.4)	.64	0.6 (0.3 to 1.3)	.21	0.6 (0.3 to 1.3)	.21
>12–23 mo	Ref	–	Ref	–	Ref	–	Ref	–
24+ mo	0.7 (0.4 to 1.0)	.07	0.7 (0.5 to 1.1)	.11	0.8 (0.5 to 1.4)	.53	0.8 (0.5 to 1.3)	.33
Female	0.8 (0.6 to 1.2)	.27	0.8 (0.6 to 1.2)	.33	0.9 (0.6 to 1.5)	.73	0.9 (0.6 to 1.5)	.73
HIV-infected^f^	Not estimable^a^		Not estimable		4.3 (3.4 to 5.5)	<.001	5.4 (3.6 to 8.1)	<.001
HIV-exposed uninfected^g^	1.3 (0.7 to 2.1)	.40	1.0 (0.6 to 1.7)	.97	0.8 (0.4 to 1.9)	.66	0.9 (0.4 to 2.2)	.86
Mid-upper arm circumference <12.5 cm	2.0 (1.3 to 3.2)	.003	1.3 (0.8 to 2.3)	.34	1.5 (0.7 to 3.1)	.33	1.7 (0.8 to 3.7)	.21
Stunting (HAZ < –2)^h^	1.0 (0.6 to 1.6)	.94	0.9 (0.6 to 1.5)	.80	0.8 (0.4 to 1.5)	.49	0.9 (0.5 to 1.6)	.64
Wasting (WHZ < –2)^i^	1.6 (1.1 to 2.5)	.03	1.5 (1.0 to 2.2)	.06	1.6 (0.9 to 2.7)	.10	1.6 (0.9 to 2.8)	.11
Currently breastfeeding (if <24 mo)^j^	1.1 (0.7 to 1.9)	.69	1.3 (0.7 to 2.3)	.40	1.6 (0.7 to 3.6)	.28	1.6 (0.7 to 3.6)	.29
No. of mo exclusively breastfed^k^	1.0 (0.9 to 1.1)	.65	0.9 (0.8 to 1.0)	.18	0.9 (0.8 to 1.1)	.21	0.9 (0.8 to 1.1)	.32
Moderate to severe diarrhea	1.0 (0.7 to 1.5)	.89	1.0 (0.6 to 1.4)	.81	1.6 (1.0 to 2.5)	.04	1.6 (1.0 to 2.5)	.04
No. of loose stools in the last 24 h	1.0 (0.9 to 1.1)	.40	1.0 (0.9 to 1.1)	.77	1.0 (0.9 to 1.1)	.63	1.0 (0.9 to 1.1)	.87
Duration of diarrhea, d	1.2 (1.1 to 1.4)	.01	1.1 (1.0 to 1.3)	.17	1.2 (1.0 to 1.5)	.03^a^	1.4 (1.1 to 1.6)	.001
Caregiver characteristics								
Age								
<20 y	2.2 (1.4 to 3.5)	.001	2.0 (1.3 to 3.1)	.002^a^	2.4 (1.2 to 4.7)	.01	2.5 (1.3 to 5.0)	.01
20–29 y	Ref	–			Ref	–	Ref	–
30+ y	1.1 (0.7 to 1.7)	.61	1.3 (0.9 to 1.9)	.22	1.6 (1.0 to 2.6)	.08	1.4 (0.9 to 2.4)	.15
HIV-infected^l^	1.2 (0.7 to 2.0)	.62	0.9 (0.5 to 1.6)	.76	1.1 (0.6 to 2.2)	.78	1.2 (0.6 to 2.6)	.55
HIV-uninfected	Ref	–	Ref	–	Ref	–	Ref	–
HIV-infected on HAART	1.1 (0.5 to 2.3)	.86	0.9 (0.4 to 1.9)	.76	0.9 (0.3 to 2.4)	.78	0.9 (0.3 to 2.5)	.82
HIV-infected not on HAART	1.2 (0.6 to 2.5)	.57	1.0 (0.5 to 1.9)	.91	1.3 (0.6 to 3.1)	.52	1.6 (0.6 to 4.0)	.32
BMI <18.5 kg/m^2^	0.9 (0.3 to 3.0)	.86	0.9 (0.3 to 3.0)	.82	1.2 (0.4 to 4.1)	.74	1.2 (0.4 to 4.1)	.74
Diarrhea in last 14 d	1.9 (1.2 to 2.9)	.01	2.2 (1.3 to 3.4)	.001	1.1 (0.5 to 2.4)	.86	1.0 (0.5 to 2.3)	.97

Abbreviations: BMI, body mass index; HAART, highly active antiretroviral therapy; HAZ, height-for-age z-score; WHZ, weight-for-height z-score.

^a^Not estimable because neither of the two HIV-infected children had *Cryptosporidium* detected.

^b^Total 77 child *Cryptosporidium* detections used.

^c^Total 57 caregiver *Cryptosporidium* detections used.

^d^Multivariable model adjusts for child age and center.

^e^Multivariable model adjusts for caregiver age and center.

^f^n = 241.

^g^n = 238.

^h^n = 236.

^i^n = 234.

^j^n = 119.

^k^n = 230.

^l^n = 240.

### Risk Factors for Caregiver *Cryptosporidium*

Risk factors for detection of *Cryptosporidium* among caregivers included *Cryptosporidium* infection in their child (aPR, 2.0; 95% CI, 1.3 to 3.1; *P = *.003) and livestock ownership: cow ownership vs none (aPR, 3.1; 95% CI, 1.4–7.0; *P = *.007) and livestock other than cows vs none (aPR, 2.6; 95% CI, 1.1–6.3; *P = *.03). Unprotected water source was not associated with caregiver *Cryptosporidium* detection. Consistent with other results, caregiver age and child diarrhea severity and duration were associated with detection of *Cryptosporidium* among caregivers, while malnutrition in the caregiver or child was not. Caregivers of children with MSD were one and a half times as likely to be positive for *Cryptosporidium* (aPR, 1.6; 95% CI, 1.0 to 2.5; *P = *.04), and 1 additional day of child diarrhea duration was associated with a 30% increased prevalence of caregiver *Cryptosporidium* infection (aPR_per day_, 1.4; 95% CI, 1.1 to 1.6; *P = *.001). Caregiver HIV infection was not associated with caregiver *Cryptosporidium* infection; however, HIV infection in the child was associated with caregiver *Cryptosporidium* detection (aPR, 5.4; 95% CI, 3.6 to 8.1; *P* < .001) (Table 4). Two children in the study were HIV positive without detection of a *Cryptosporidium* infection, and both caregivers were positive for PCR detection of *Cryptosporidium* and were HIV-infected and not on HAART.

## DISCUSSION

In this cross-sectional study of children presenting to Kenya hospitals with acute diarrhea and their accompanying caregivers, we found that *Cryptosporidium* prevalence was high. The presence of *Cryptosporidium* infection in caregivers was a risk factor for infection in their children, and similarly the presence of *Cryptosporidium* in children was a risk factor for infection in their caregiver. Diarrhea and diarrhea severity were also associated with child and caregiver *Cryptosporidium* infections, respectively, consistent with possible person-to-person transmission within the child–caregiver pairs.

We describe a prevalence (~30%) of *Cryptosporidium* infection among children with diarrhea on the higher end of the range reported in other studies conducted in Sub-Saharan Africa (13–32%) [12, 24–26]. In this study, over a third of caregivers of *Cryptosporidium*-infected children with diarrhea also had *Cryptosporidium*. This prevalence (35%) is within the range of 2 recent studies evaluating *Cryptosporidium* infections in household contacts of children with *Cryptosporidium* in Bangladesh and in a multicountry study in Sub-Saharan Africa (Gabon, Ghana, Madagascar, and Tanzania) reporting prevalence rates of 51% and 31%, respectively [12, 27]. Taken together, these studies highlight that *Cryptosporidium* is likely present in 1 or more additional household members during episodes of *Cryptosporidium* diarrhea in a child, which presents challenges for prevention and control strategies.

Studies conducted in Norway [9], Brazil [28], Bangladesh [27], and, most recently, in a multicountry study in Gabon, Ghana, Madagascar, and Tanzania [12] have observed evidence of person-to-person transmission, further supporting our associative findings. *Cryptosporidium* is characteristically highly infectious and associated with persistent diarrhea [5, 28, 29] and continued *Cryptosporidium* oocyst shedding after diarrhea ceases [30, 31], providing a long duration of infectivity. We found diarrhea and diarrhea severity to be risk factors for child and caregiver *Cryptosporidium* infection, respectively, further substantiating a possible person-to-person transmission. Contact with a person with diarrhea has previously been noted as a risk factor for *Cryptosporidium* infection [32], particularly in outbreak investigations [9, 33]. While as much as 50% of *Cryptosporidium* infection may be asymptomatic, *Cryptosporidium* infection with diarrhea is more infectious, with increased exposure to the parasite through symptoms and higher parasitic burden relative to asymptomatic infection [5].

Person-to-person transmission is unlikely to be the only source of *Cryptosporidium* infection. We found livestock ownership and unprotected water source to be important risk factors for caregiver and child *Cryptosporidium* infection, respectively. Livestock ownership and contaminated water source are well documented as sources of transmission for *Cryptosporidium* in low-resource settings, although they are inconsistently identified as significant risk factors for *Cryptosporidium* infection in individual studies [6, 25, 29, 34]. Contaminated water is often associated with *Cryptosporidium* outbreaks [11, 32, 35, 36]; however, the source of sustained endemic transmission remains unclear and may include a combination of contaminated water, zoonotic, and person-to-person transmission depending on the setting and population.

*Cryptosporidium* infection is more common and more severe among immunocompromised hosts, particularly adults with HIV and young children with malnutrition [5, 37]. However, in this study, neither HIV infection in caregivers nor malnutrition among children was significantly associated with detection of *Cryptosporidium*. The lack of association may, in part, be due to the relatively small number of HIV-infected caregivers not on antiretroviral therapy (ART) and children with acute malnutrition, which limited our statistical power to detect an association. The use of antiretrovirals for treatment and improved immune function of people with HIV appears to have reduced the prevalence of *Cryptosporidium* infection among persons with HIV [38, 39]. Our results did show that 100% of the 2 HIV-infected children had caregivers with PCR-detected *Cryptosporidium* but, little can be drawn from such small numbers.

The concordance between the point-of-care ELISA test and PCR results was consistent with previous research. PCR is known to be more sensitive than most immunoassays, often detecting small quantities of *Cryptosporidium* DNA in diarrheal episodes likely caused by another pathogen [40]. In our study, *Cryptosporidium* was detected in samples from 2 asymptomatic caregivers by immunoassay and not by PCR. The 2 immunoassay positives may have been false positives or false negatives by PCR, both of which have been reported, although not frequently, in the literature [21, 40, 41].

This study has several important limitations. Due to the cross-sectional nature of the study design and lack of genotyping, we were unable to confirm transmission of *Cryptosporidium* infection within the child–caregiver pair, or the direction of the transmission. Further, household members other than the child and caregiver were not included in the study; as such, we are unable to make inferences about the caregiver–child pair relationship in context of other persons living in the household. Additional research should assess the importance of the primary caregiver relative to other household members as a source of *Cryptosporidium* infection for young children. Lower numbers of children with acute malnutrition and immunocompromised caregivers would explain our inability to detect a significant association between these host factors and *Cryptosporidium* infection.

The results of this study suggest that the child–caregiver dyad should be further explored as a source of household person-to-person transmission. If transmission between young children and their primary caregivers, which in this context is mostly biological mothers, is common, effective disease control strategies may need to focus on prevention and treatment of infection among caregivers to effectively reduce *Cryptosporidium-*associated disease, malnutrition, and mortality in children.
